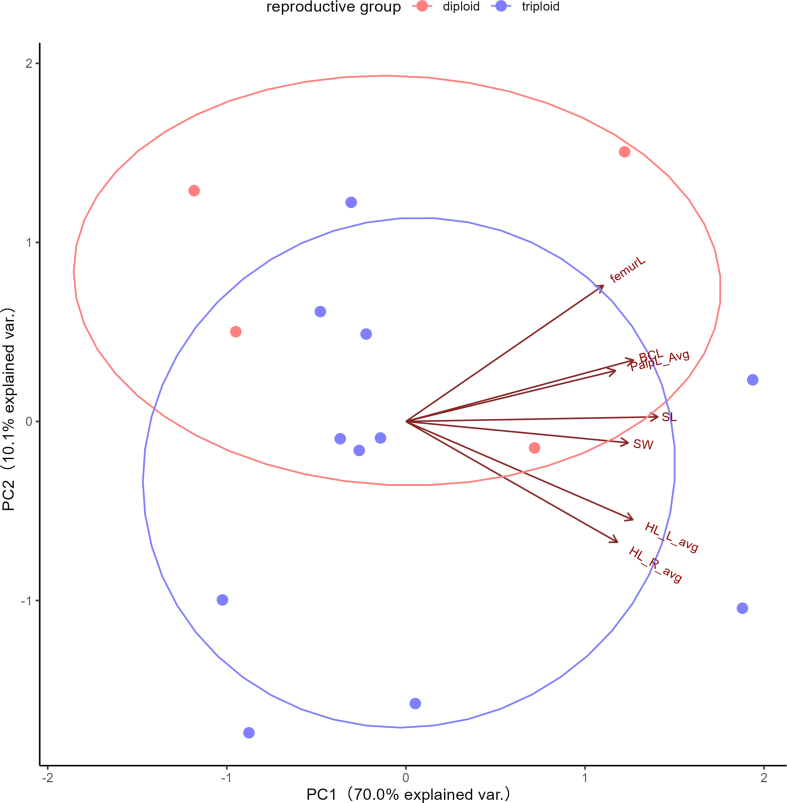# *COI* barcoding can distinguish bisexual and parthenogenetic populations of *Haemaphysalis longicornis* in Japan: Revisiting methods with SNP analysis as another possible method

**DOI:** 10.1016/j.ijppaw.2025.101083

**Published:** 2025-05-15

**Authors:** Mizue Inumaru, Kentaro Itokawa, Ryo Matsumura, Kyoko Sawabe, Mamoru Watanabe, Haruhiko Isawa, Shinji Kasai, Yukiko Higa

**Affiliations:** a Department of Medical Entomology, National Institute of Infectious Diseases, Toyama 1-23-1, Shinjuku, Tokyo, 162-8640, Japan; b Graduate School of Agriculture, Meiji University, Higashimita 1-1-1, Tama, Kawasaki, Kanagawa, 214-8571, Japan; c Graduate School of Agricultural and Life Sciences, University of Tokyo, Yayoi 1-1-1, Bunkyo, Tokyo, 113-8657, Japan

**Keywords:** *COI* barcoding, *Haemaphysalis longicornis*, Morphometric analysis, Parthenogenesis, SNP genotyping

## Abstract

*Haemaphysalis longicornis*, the Asian long-horned tick, is an important vector for various infectious diseases, such as severe fever with thrombocytopenia syndrome (SFTS) and Japanese spotted fever. In this species, a triploid parthenogenetic reproductive form occurs along with a diploid bisexual form. Several approaches have been used to distinguish these two groups, including the presence/absence of males in the population, karyotyping, flow cytometry, and most recently, mitochondrial phylogeny. Mitochondrial gene (*COI*) barcoding has also been casually used, although its validity has not been investigated. In the present study, the validity of *COI* barcoding, genotyping nuclear markers (SNPs), and morphometrics was evaluated for distinguishing the reproductive forms of *H. longicornis* in Japan. Ticks were collected using the flagging method at two locations in Hyogo, Japan. DNA was extracted from ticks after photography, which was used for morphometric measurements. The DNA was used for *COI* barcoding by direct sequencing and genotyping SNPs in the nuclear genome. The resulting *COI* haplotypes were clustered into two distinct haplogroups, which represented different ploidy levels, corresponding to the different reproductive groups. Genotypes of nuclear SNPs supported that the individuals from each mitochondrial haplogroup belonged to distinct reproductive populations with different ploidy levels. Meanwhile, although significant differences were observed in multiple morphometric characteristics between these reproductive groups, large overlaps were generally evident in the distribution, indicating that morphological identification is not sufficient to distinguish the reproductive groups. This study suggested for the first time that *COI* barcoding and SNP genotyping are both convenient and reliable methods to distinguish the two reproductive forms of *H. longicornis* in Japan.

## Highlights

• Triploid (parthenogenetic) haplogroup formed monophyletic clade.
• SNP genotyping and *COI* barcoding effective to distinguish reproductive forms.
• *COI* region by itself well represents mitochondrial genome.
• Morphometrics insufficient to accurately distinguish reproductive forms.

## Graphical abstract

**Figure:**
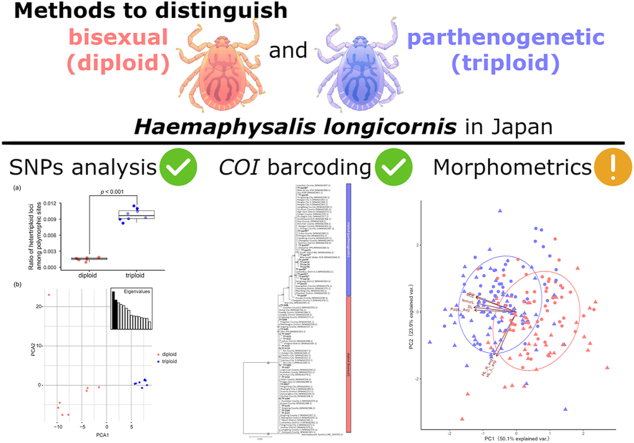

## 1 Introduction

*Haemaphysalis longicornis*, known as the Asian long-horned tick, is well-known for its ability to transmit severe fever with thrombocytopenia syndrome virus (SFTSV), *Rickettsia japonica* which causes Japanese spotted fever, along with several other pathogens (Hoogstraal et al., 1968; Zhang et al., 2022; Zhao et al., 2020). The species is native to eastern Asia, including China, Japan, the Republic of Korea, and parts of Russia (Egizi et al., 2020; Zhao et al., 2020), although its distribution has extended to Australia, New Zealand, other Pacific islands, and most recently the United States (Egizi et al., 2020; Hoogstraal et al., 1968). *Haemaphysalis longicornis* is one of the few tick species that exhibit triploid parthenogenetic forms (i.e., females can reproduce without mating with males) along with normal diploid bisexual forms (Kitaoka, 1961; Oliver and Bremner, 1968). Distinguishing between these forms is crucial for understanding the potential differences in population dynamics, evolutionary adaptability, and their role in disease transmission (Zhang et al., 2022).

Classically, the presence or absence of males has been generally used to classify the two reproductive forms at the population level. If males are present, the population is considered bisexual; otherwise, the population is considered parthenogenetic. However, this approach does not consider the possibility of sympatric distribution of both reproductive forms. At the individual level, laborious laboratory feeding is necessary for both checking the sex bias of the offspring (Chen et al., 2012; Kitaoka, 1961) and karyotype analysis, using microscopy (Chen et al., 2014; Oliver et al., 1973) or flow cytometry (Chen et al., 2014; Zhang et al., 2022). These techniques also require specialized skills and facilities. Some studies have included morphometric measurements because parthenogenetic females are slightly larger in size compared with bisexual females (Chen et al., 2012; Herrin and Oliver, 1974). Recently, Zhang et al. (2022) demonstrated that there are two distinct sister lineages in *H. longicornis* mitochondria, and individual karyotypes distinguished by flow cytometry corresponded to these mitochondrial lineages. Therefore, mitochondria may serve as a useful genetic marker to distinguish different ploidy levels corresponding to the reproductive forms of *H. longicornis*. Furthermore, studies have used the partial *COI* gene widely used for barcoding (Egizi et al., 2020; Morii et al., 2025), although the validity of this region has not been established.

In the study by Zhang et al. (2022), most of the specimens were from China, and only three specimens of *H. longicornis* from Japan were included for the mitochondrial phylogenetic study. In the present study, we used SNP genotypes from the nuclear genome to confirm the existence of reproductively isolated populations with different ploidy levels in *H. longicornis* from Japan. We then tested morphometric measurements and *COI* barcoding to determine the ability of these methods to differentiate those groups. Importantly, we explored the possibility that only the sequencing of the barcoding region of the mitochondrial *COI* gene, which is commonly used for species identification, retains sufficient diversity to distinguish the reproductive forms.

## 2 Materials and methods

### 2.1 Tick collection and species identification

Ticks were collected by the flagging method at two locations in Toyooka, Hyogo, Japan (Igadani: 35°35′N 134°46′E; Koryuji: 35°30′N 134°58′E; Fig. 1) on May 14, 2022. At these locations, it has been previously confirmed that *H. longicornis* is the dominant species (Yamauchi et al., unpublished). 1702 *H. longicornis* were morphologically identified using a stereo microscope (Olympus-SZ61, Japan) and a light microscope (Nikon Eclipse Ci, Japan). Of the collected individuals, 299 *H*. *longicornis* were randomly selected (272 nymphs, 18 adult females, 9 adult males; Table 1). Morphological features were photographed using an Olympus DP28 camera and a Leica MC 170 HD camera. The ticks were maintained at 4 °C until further processing.

**Fig. 1:**
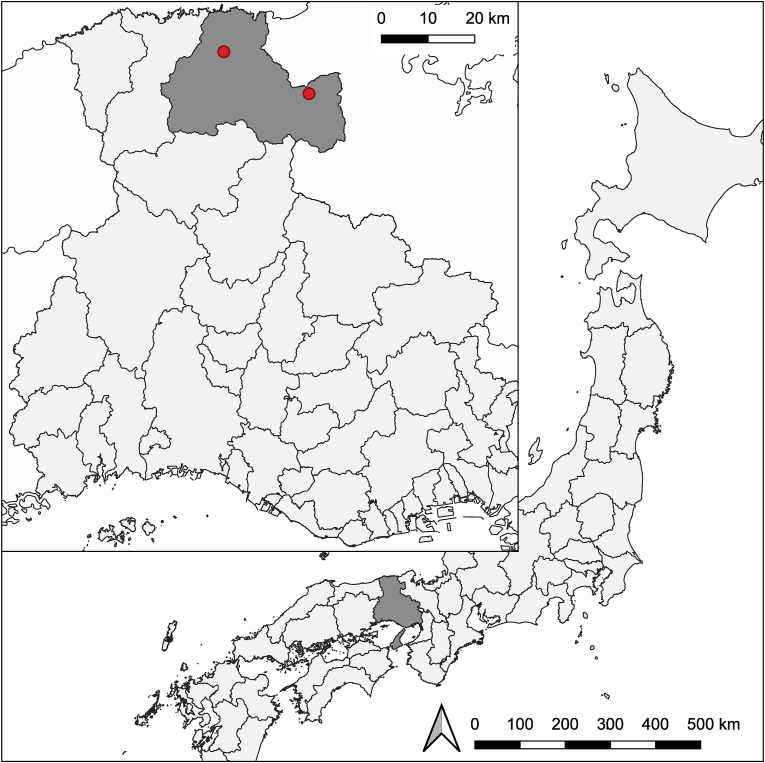
Map of the sampling locations. In the map of Japan, Hyogo Prefecture, where sampling took place, is shown in dark gray. The inset map shows the locations (red dots; left: Igadani; right: Koryuji) and city (Toyooka; dark gray) of sampling.

**Table 1:** *COI* haplotypes of *Haemaphysalis longicornis* found in this study.

Haplotypes	Koryuji					Igadani		total
	nymph	female	male	total		nymph		
TY-par01	23	7	0	30		6		36
TY-par02	3	0	0	3		5		8
TY-par03	2	0	0	2		0		2
TY-par04	16	3	0	19		1		20
TY-par05	19	0	0	19		8		27
TY-par06	28	1	0	29		15		44
TY-par07	1	1	0	2		0		2
TY-par08	9	1	0	10		1		11
TY-par09	2	0	0	2		3		5
TY-par10	1	0	0	1		0		1
TY-par11	10	0	0	10		2		12
TY-par12	2	0	0	2		0		2
TY-par13	4	0	0	4		0		4
TY-par14	1	0	0	1		0		1
TY-par15	0	0	0	0		2		2
TY-par16	0	0	0	0		2		2
subtotal	121	13	0	134		45		179

TY-bi01	37	1	4	42		18		60
TY-bi02	7	1	1	9		1		10
TY-bi03	4	0	0	4		4		8
TY-bi04	4	0	0	4		3		7
TY-bi05	2	0	0	2		0		2
TY-bi06	1	0	0	1		0		1
TY-bi07	3	2	0	5		2		7
TY-bi08	3	1	1	5		2		7
TY-bi09	1	0	1	2		0		2
TY-bi10	2	0	2	4		3		7
TY-bi11	1	0	0	1		0		1
TY-bi12	1	0	0	1		0		1
TY-bi13	1	0	0	1		0		1
TY-bi14	1	0	0	1		0		1
TY-bi15	1	0	0	1		0		1
TY-bi16	0	0	0	0		4		4
subtotal	69	5	9	83		37		120

Total	190	18	9	217		82		299

No. of triploid haplotypes	14	5	0	14		10		16
No. of diploid haplotypes	15	4	5	15		8		16
Total no. of haplotypes	29	9	5	29		18		32

### 2.2 DNA extraction

DNA was extracted from either whole tick bodies (n = 77) or two legs (n = 222) using different methods. For whole body extraction, ticks were placed in 2 ml tubes containing 40 μl of DNA/RNA shield™ (Zymo Research) and a stainless bead (φ5 mm; Askul Corp., Tokyo, Japan). The ticks were crushed with TissueLyser II (Qiagen, Tokyo, Japan) at 15 Hz for 1.5 min followed by 18 Hz for 1 min. After homogenization, 2–6 μl of proteinase K was added to each sample, which was incubated at 60 °C with shaking at 14 RPM overnight in a Hybridization Incubator HB-80 (Taitec, Saitama, Japan). The samples were homogenized again at 18 Hz for 1 min, and DNA was extracted using the Quick-DNA/RNA™ MagBead Kit (Zymo Research). The purified DNA was eluted into 50 μl of low-TE (10 mM Tris-HCl and 0.1 mM EDTA, pH 8.0). For some ticks, DNA was crudely extracted from the legs using a modified alkaline extraction method (Rudbeck and Dissing, 1998). The legs were first removed from the body using a sterile scalpel and tweezers, which were sterilized between each tick. The removed legs were placed into 0.2 ml tubes containing two zirconium beads (φ2 mm, BMS, Tokyo, Japan) and 5 μl of 0.2 M NaOH, and the samples were homogenized with TissueLyser II (Qiagen) at 25 Hz for 1 min. After incubation for 10 min at 70 °C, 20 μl of a buffer mixture containing 90 mM Tris-HCl and 1.25 mM EDTA was added to each sample. The DNA samples were stored at −20 °C until further processing.

### 2.3 Sequencing the *COI* barcoding region

PCR targeting the partial tick *COI* gene was performed with the dgLCO1490 (tailed with the M13 forward sequence) and dgHCO2198 (Meyer, 2003) primers, which were modified from the widely used universal primer set (Folmer et al., 1994). Each reaction mixture included 0.4 μM of deoxynucleotide triphosphate (dNTP), 1x KOD FX buffer (Toyobo, Japan), 0.2 U KOD FX (Toyobo), 0.25 μM of each primer, and 1 μl of template DNA for a total volume of 10 μl. One negative control containing distilled water instead of DNA was included in each PCR reaction. After an initial 2 min at 94 °C, three cycles at 98 °C 10 s, 50 °C 30 s, and 68 °C 45 s were carried out, followed by 37 cycles under the same conditions, except the annealing temperature was 53 °C instead of 50 °C. A final incubation at 68 °C for 5 min was performed. Amplification was confirmed by microchip electrophoresis using MultiNA (Shimadzu Co, Kyoto, Japan). The PCR products were purified using Agencourt AMPure XP beads (Beckman Coulter, Tokyo, Japan) and sent to Azenta (Tokyo, Japan) for sequencing using the M13 forward sequencing primer. The resulting sequences were compared with the available sequences in the GenBank database using the BLAST algorithm (Madden, 2013).

### 2.4 Phylogenetic analysis

The primer sequence was trimmed from the *COI* sequences as well as low-quality portions using ATGC software (GENETYX, Tokyo, Japan). The full mitochondrial DNA sequences described by Zhang et al. (2022) (MW642369.1–407.1) were downloaded as reference panels. The *COI* barcoding region was extracted and multi-aligned with the *COI* gene sequences obtained in the present study using GENETYX ver. 13 (GENETYX, Tokyo, Japan). A phylogenetic tree for the *COI* barcoding sub-region was constructed using the maximum-likelihood (ML) method with 1000 bootstrap tests in MEGA X (Kumar et al., 2018). The Tamura-3-parameter model with Gamma distribution was selected as the best fit model by MEGA X. For the full-length mitochondrial sequence tree from the data of Zhang et al. (2022), multiple alignment was achieved by MAFFT v7.520 with the full-length mitochondrial DNA sequences of *H. flava* (NC_005292.1) (Shao et al., 2004) and *H. hystricis* (NC_039765.1) (Tian et al., 2019) as outgroups. Poorly aligned regions were trimmed using TrimAl v1.4.1 (Capella-Gutiérrez et al., 2009) in “strict” mode. IQ-Tree v2.2.6 (Hoang et al., 2018; Kalyaanamoorthy et al., 2017; Minh et al., 2020) was used to infer the best-fit substitution model, construct an ML tree, and conduct 1000 ultrafast-bootstrap tests.

### 2.5 RADseq

Among the genomic DNA extracted from whole bodies, eight samples were randomly selected from each of the diploid and triploid mitochondrial haplogroups. RADseq (Miller et al., 2007) was performed using a method similar to ezRADseq (Toonen et al., 2013). For a 20 μl reaction, 25 ng of DNA was digested with 1 U of *EcoRV-HF* (New England Biolabs, Tokyo, Japan) in rCutSmart buffer (New England Biolabs). The digestions were cleaned and ligated with IDT for Illumina-TruSeq DNA UD index adapters (Illumina, Tokyo, Japan) using a blunt/TA ligase master mix (New England Biolabs). The adapter-ligated DNA was incubated at 70 °C for 15 min to inactivate the ligase, and all indexed DNA was pooled into a single tube. Following purification on Agencourt AMPure XP beads (Beckman Coulter), the pooled DNA was electrophoresed on a 2 % agarose gel in 1x TAE buffer. A gel block containing fragments between 450 and 650 bp (insert size 300 to 400 bp) was cut, and the DNA was purified using the MonoFas DNA purification kit (ANIMOS Inc., Saitama, Japan). The library concentrations were confirmed by qPCR (QuantStudio 1; Thermo Fisher Scientific K.K., Tokyo, Japan), followed by PCR to adjust the concentrations. Finally, the libraries were pooled with libraries from other projects and sequenced on a Next Generation Sequencer (NGS), the Illumina NextSeq 1000 platform (Illumina), using the P1 flow cell (2 x 150 bp). In total, 5.2 Gbp of data, ranging from 93 to 603 Mbp per individual, was obtained for 16 samples. The data were deposited in the DNA Data Bank Japan (DDBJ) under BioProject accession PRJDB20261.

### 2.6 SNP genotyping, clustering, and counting of heterotriploid genotypes

The NGS reads were mapped to the *H. longicornis* reference genome ASM1333976v1 (Jia et al., 2020) with BWA-MEM in BWA ver. 0.7.17 (Li, 2013) and sorted using SAMtools ver. 1.14 (Danecek et al., 2021). The resulting BAM files were subjected to variant calling using gstacks and population in STACKS ver. 2.68 (Catchen et al., 2011). The minimum depth to call a genotype was set at 15, and the minimum percentage of individuals across populations required to process a locus was set at 80 %. In total, 10,806 RAD loci were identified, and 2204 of them were polymorphic and passed the filter. The haplotype genotype was used for principal component analysis (PCA) using Adegenet ver. 2.1.10 (Jombart, 2008) in R ver. 4.3.2 (R Core Team, 2023). SNPs within the RAD loci detected by the STACKS analysis were re-genotyped using Freebayes ver. 1.3.6 (Garrison and Marth, 2012) with the option “-p 3” because STACKS does not support polyploid genotypes. The number of heterotriploid genotypes (containing three different alleles at a single locus in a single individual, e.g., 0/1/2, 0/1/3, etc.) was counted for each individual using R. The genotypes were considered for counting only if the variant quality value (QUAL) was >200, the mean map qualities of reads (MQM) > 50, and the mean depth per individual >100 to avoid potential repetitive regions.

### 2.7 Morphometric measurements

The following features were measured per individual: scutum (length and width; between the longest/widest points), palp length (between the posterior-most point of the external margin of segment 2 and the anterior-most point of the external margin of segment 3), basis capituli length (between the central points of the external margins), femur length of leg I (between the longest points of each end), and length of the internal spur of coxa I (between the dense base, distinguished by transmitted light observation, and posterior tip of the coxa) (Additional file: Fig. S1). Measurements were taken using photographs and the software ImageJ 1.53k (National Institutes of Health, MD, USA). Measurements were compared between the ploidy groups and also between the locations for the nymphs using one-way or two-way ANOVA and PCA in the software R. The packages “car” and “stats” were used for statistical analyses (Fox and Weisberg, 2011; R Core Team, 2023). Note that the ploidy groups were based on the *COI* barcoding results.

## 3 Results

**Fig. 2:**
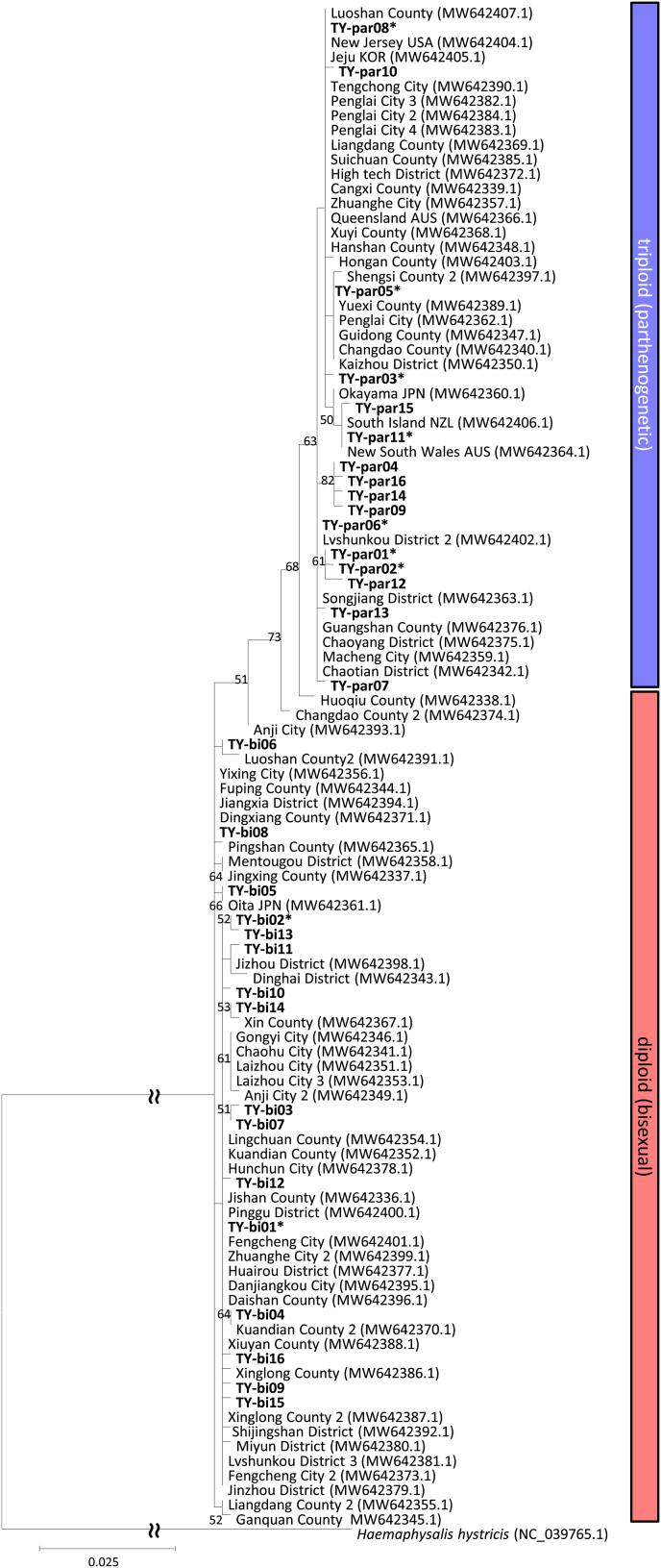
Maximum likelihood tree for the *COI* sequences (659 bp) of *Haemaphysalis longicornis*, constructed using the Tamura-3-parameter model. The diploid (bisexual) and triploid (parthenogenetic) clusters are marked based on the reference haplotypes from Zhang et al. (2022), which are included in the tree. *Haemaphysalis hystricis* was used as an outgroup. Haplotypes from the present study are shown in bold. Haplotypes from individuals subjected to SNP analysis are marked with asterisks (∗).

A total of 32 unique *COI* haplotypes were confirmed from 299 individuals (Table 1, Fig. 2). Phylogenetic analysis clustered the 16 haplotypes obtained in this study (TY-par01 to −16; Genbank accession numbers LC860032-LC860047) into a monophyletic clade containing the haplotypes associated with triploidy (parthenogenetic) based on Zhang et al. (2022). The other 16 haplotypes obtained in this study (TY-bi01 to −16; Genbank accession numbers LC860048-LC860063) and the haplotypes associated with diploidy (bisexual) from Zhang et al. (2022) formed a paraphyletic clade of the monophyletic triploid clade (Fig. 2). Both haplogroups included nymphs and females examined in this study, whereas all males were placed in the diploid haplogroup. Haplotypes in both clades existed at both sampling sites. Notably, the ML tree generated in the present study (Fig. 2) highly resembled that of the mitochondrial genomes in Zhang et al. (2022) when an outgroup was included (Additional file: Fig. S2). For example, all haplotypes from Zhang et al. (2022) were placed into the same clade for both trees.

**Fig. 3:**
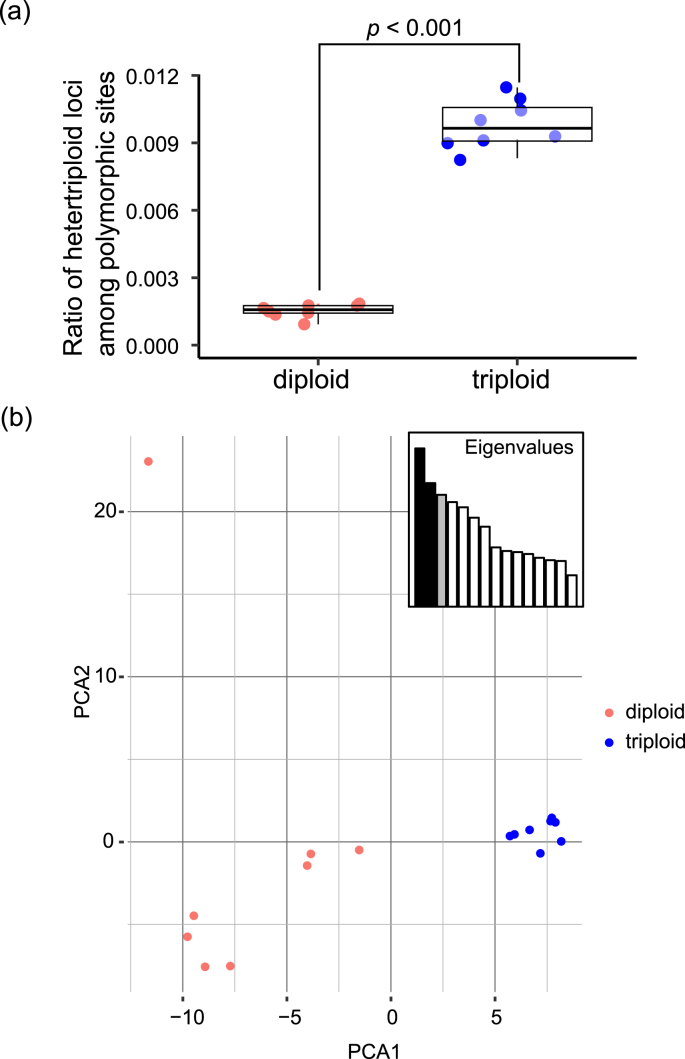
(a) The proportion of the number of heterotriploid SNP/MNP loci among all variable loci for the bi and par *COI* haplogroups. The *p*-value was calculated by the Wilcoxon rank sum test. (b) Plot of the first and second components of the PCA for the SNP/MNP genotypes. The red and blue dots indicate the bi and par *COI* mitochondrial haplogroups, respectively.

Fifteen nymphs and one male were randomly selected and used for SNP analysis (seven and two *COI* haplotypes from triploid and diploid haplogroups, respectively). The *COI* haplotypes of these individuals were TY-par01 (*n* = 1), TY-par02 (*n* = 1), TY-par03 (*n* = 1), TY-par05 (*n* = 1), TY-par06 (*n* = 1), TY-par08 (*n* = 2), and TY-par11 (*n* = 1), TY-bi01 (*n* = 7), and TY-bi02 (*n* = 1) (Fig. 2). Of the individuals subjected for SNP analysis, the number of heterotriploid genotypes (containing three different alleles at a single locus in a single individual) was significantly higher (*p* < 0.001 in Wilcoxon rank sum exact test) in the individuals with triploid haplotypes compared with those containing diploid haplotypes (Fig. 3a). In addition, PCA clustered the SNP genotypes (genotyped as diploid) of the two haplogroups into distinct clusters at the first axis (Fig. 3b).

**Fig. 4:**
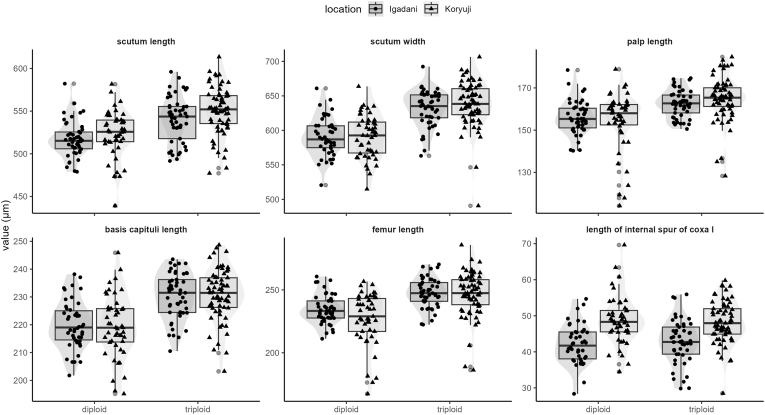
Measurements of six morphological features of the *H. longicornis* nymphs by location and reproductive group. Note that the reproductive groups were separated by *COI* haplotypes. Brackets and asterisks (∗∗∗) show significant differences (*p* < 0.05) between reproductive groups (all except length of internal spur of coxa I) and locations (length of internal spur of coxa I), calculated by a two-way ANOVA. See Additional file: Fig. S1 for illustrations of the measured features.

**Fig. 5:**
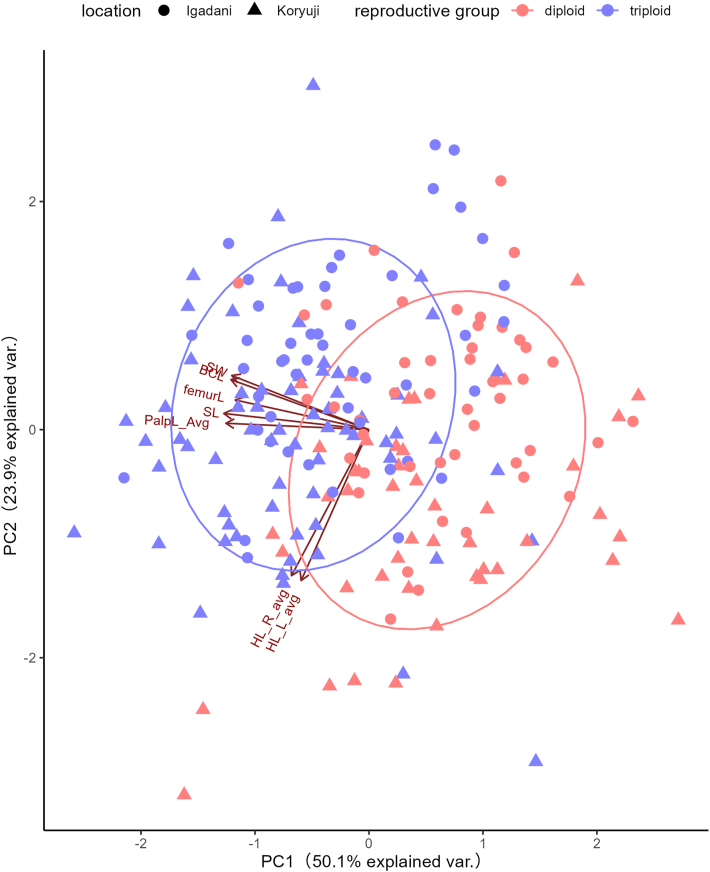
PCA of the morphological measurements of *H. longicornis* nymphs. The colors and shapes represent the reproductive groups and locations as shown in the legend.

Among the nymphs, all measurements except for the spur lengths of coxa IV were significantly different between the diploid and triploid individuals, with the triploid individuals being larger (Table 2, Fig. 4). Meanwhile, the distribution of measurement values greatly overlapped between the two groups. Additionally, note that the scutum and spur lengths of coxa IV were significantly larger in individuals collected at Koryuji compared with those at Igadani, although there were no significant differences between locations for all other measurements (Table 2, Fig. 4). Using PCA, 2 PCs explained 74.81 % of the variations (PC1 = 50.92 %, PC2 = 23.89 %; Fig. 5). Similar to the results from a two-way ANOVA, although the factor loadings for PC1 were related to all measurements except the spur lengths of coxa IV, the two reproductive groups were not clearly separated. For females, no significant differences were detected for all measurements, and there was no clear separation by PCI (Additional file: Table S1, Fig. S3).

**Table 2:** Two-way ANOVA results for the measurements of *Haemaphysalis longicornis* nymphs.

	df	sum square	F ratio	P value
Scutum length				
location	1	3895	5.79	0.02
reproductive group	1	31242	46.47	<0.01
interaction	1	167	0.25	0.62
error	203	136467		
Scutum width				
location	1	628	0.70	0.4
reproductive group	1	99054	110.43	<0.01
interaction	1	367	0.41	0.52
error	203	182083		
Palp length (average)				
location	1	1.6	0.01	0.92
reproductive group	1	3335.6	25.85	<0.01
interaction	1	124.8	0.97	0.33
error	203	26197.1		
Length of the basis capituli				
location	1	0.8	0.01	0.92
reproductive group	1	5972.5	68.82	<0.01
interaction	1	11.1	0.13	0.72
error	203	17618.2		
femur length of leg I				
location	1	845	3.28	0.07
reproductive group	1	13783	53.57	<0.01
interaction	1	539	2.10	0.15
error	203	52230		
Length of the internal spur of coxa I (average)				
location	1	1804.5	50.60	<0.01
reproductive group	1	0.3	0.01	0.93
interaction	1	21.8	0.61	0.44
error	203	7239.1

## 4 Discussion

PCA clustered the nuclear SNP genotypes of 16 selected individuals into two distinct genetic populations, each corresponding to the two mitochondrial haplogroups obtained by *COI* barcoding (“triploid” and “diploid”). Moreover, the triploid haplogroup individuals showed a significantly higher number of heterotriploid loci in their nuclear SNPs compared with those in the diploid haplogroup individuals, which confirms the presence of a genuine triploid karyotype. Thus, the SNP analysis in the present study is considered to effectively distinguish between the two ploidy levels. Furthermore, similar to the results of Zhang et al. (2022), our study indicated a clear association between the mitochondrial haplogroup and the nuclear ploidy levels of *H. longicornis*. Further investigations using this novel SNP analysis method for specimens from various regions would help to confirm the robustness of this method. As SNP analyses are becoming more and more common, this novel method will serve as a powerful addition to the previously known methods.

The ML tree of the partial *COI* barcoding region recovered a monophyletic clade including all of the haplotypes associated with triploidy in Zhang et al. (2022) and some haplotypes obtained in the present study. This suggests a single evolutionary origin of triploidy in *H. longicornis*, which is linked to the parthenogenetic reproductive form (Oliver and Bremner, 1968). Importantly, the phylogenetic tree constructed with only the *COI* barcoding region showed good concordance with the tree using the full mitochondrial DNA sequence from Zhang et al. (2022) when an outgroup was added. This indicates that the *COI* barcoding region alone, which is commonly used for species identification, contains sufficient information to distinguish the two haplogroups, and hence, the reproductive forms. The usability of the *COI* gene marker to distinguish reproductive forms was also discussed by Egizi et al. (2020), whereas the analysis contained few individuals (from laboratory colonies) whose reproductive forms were unambiguously characterized. Furthermore, Egizi et al. (2020) utilized globally-sources samples, meaning that differences among clusters could reflect population-level variations. In the present study, samples including those confirmed by SNP analysis were obtained from the same area, substantially lowering the above possibility and confirming the sensitivity of this method. A previous study in Japan uses the *COI* region to distinguish the two reproductive forms (Morii et al., 2025), although this method is based on assumptions through previous studies. This study suggests for the first time that *COI* barcoding alone can reliably distinguish the two forms of *H. longicornis* in Japan.

In addition to the molecular genetics approach, morphometric methods to distinguish the reproductive forms were evaluated. Parthenogenetic individuals are generally considered larger than bisexual individuals (Hoogstraal et al., 1968; Kitaoka, 1961). A previous study using 55 phenetic characteristics (both quantitative and qualitative) found that the reproductive forms of *H. longicornis* were distinguishable to a certain degree (Herrin and Oliver, 1974), whereas the groups were not clearly separable in many cases. Similar to previous studies (Chen et al., 2012; Herrin and Oliver, 1974), significant differences were detected in multiple morphometric measurements between the two distinct ploidy levels in the present study; however, the distribution of these values greatly overlapped. Furthermore, there were significant variations in the scutum and spur lengths between the sampling locations. Therefore, while significant differences exist in the body size between the two ploidy levels, molecular genetic methods (SNP or *COI*) were far more accurate for distinguishing them.

In Hyogo, both ploidy levels representing the reproductive forms were detected in both sampling areas. The sympatric distribution of the two reproductive forms was also reported in 18 out of 73 counties assessed in China (Zhang et al., 2022). Some older studies inferred such sympatric distribution from a skewed sex ratio (Kitaoka, 1961), but the accuracy is questionable. Methods warranted to distinguish the reproductive forms of *H. longicornis* at the individual level have been long awaited, and the use of the *COI* barcoding region will sufficiently advance research to reveal a more detailed yet accurate picture of the distribution of the forms.

## CRediT authorship contribution statement

**Mizue Inumaru:** Writing – original draft, Methodology, Investigation, Formal analysis, Data curation, Conceptualization. **Kentaro Itokawa:** Writing – original draft, Methodology, Formal analysis, Data curation, Conceptualization. **Ryo Matsumura:** Investigation. **Kyoko Sawabe:** Investigation. **Mamoru Watanabe:** Investigation. **Haruhiko Isawa:** Investigation. **Shinji Kasai:** Supervision, Funding acquisition. **Yukiko Higa:** Writing – review & editing, Supervision, Funding acquisition.

## Data statement

All data generated in the present study are reported in this article.

## Funding

This work was supported by the 10.13039/100009619Japan Agency for Medical Research and Development (Grant nos. JP23fk0108613, JP23fk0108625, JP24fk0108693, and JP23wm0225030).

## Declaration of interest

All authors declare no conflict of interest associated with this study.

## Figures and Tables

**figs1:**
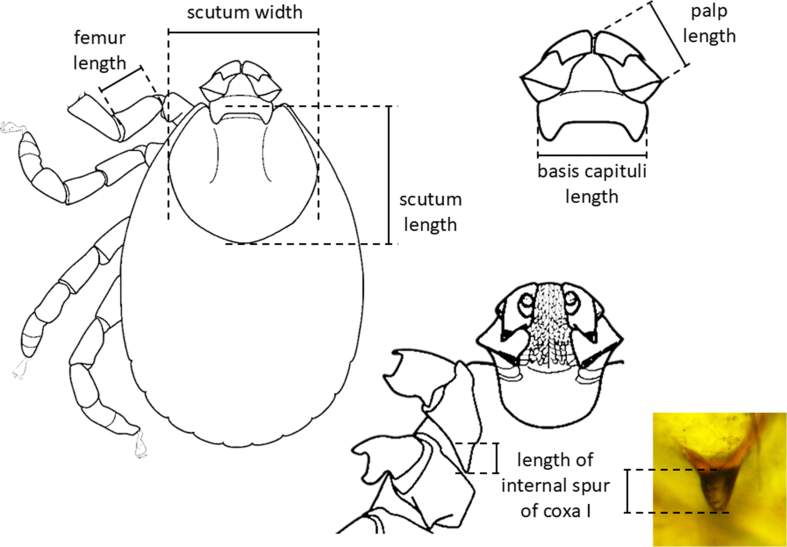


**figs2:**