# Supplementary material for: COI barcoding can distinguish bisexual and parthenogenetic populations of Haemaphysalis longicornis in Japan: Revisiting methods with SNP analysis as another possible method
Source: Int J Parasitol Parasites Wildl. 2025 May 15;27:101083. doi: 10.1016/j.ijppaw.2025.101083 (PMC12143733; doi:10.1016/j.ijppaw.2025.101083)
Supplement: Multimedia component 2 [file mmc2.pdf]

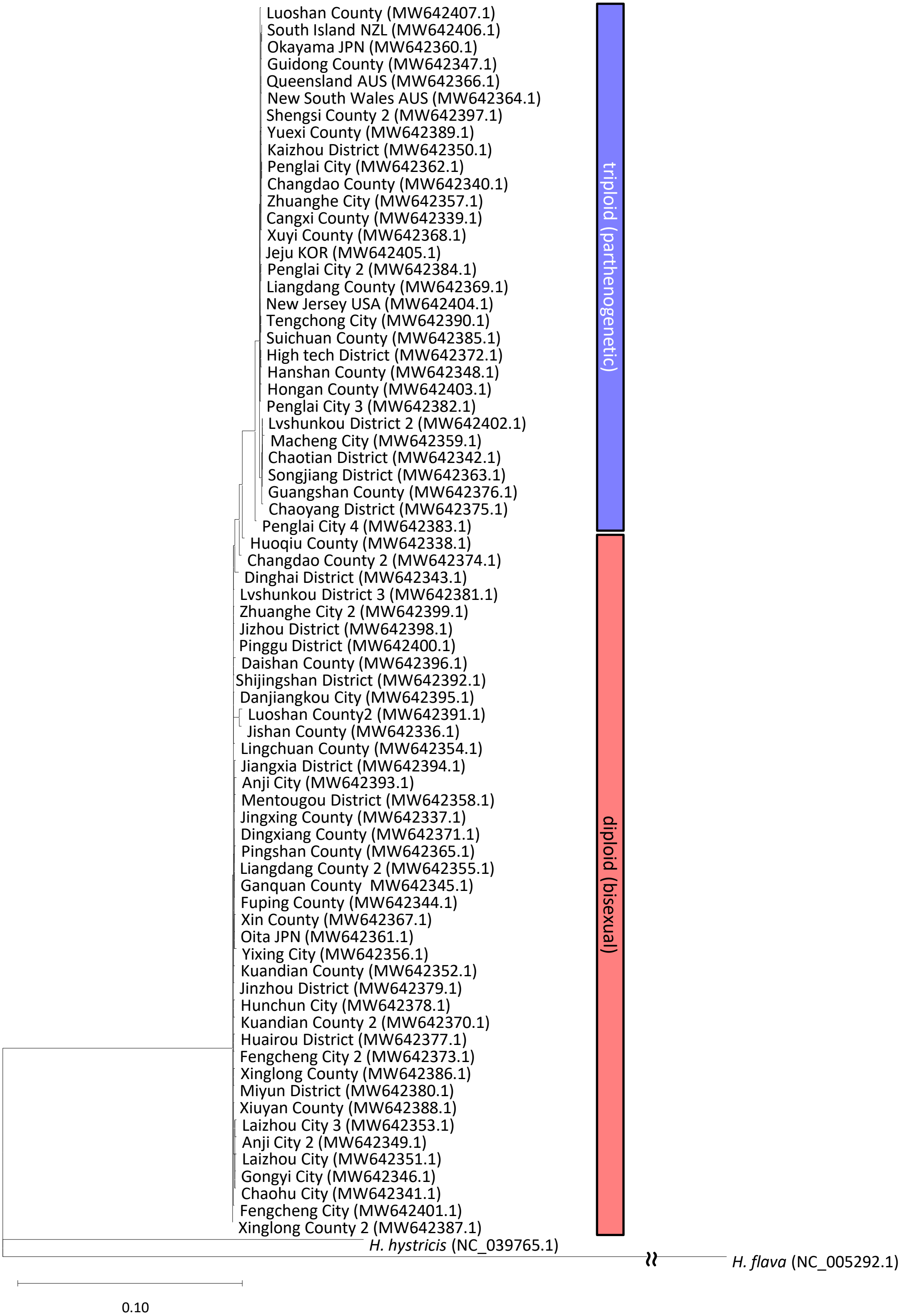
Luoshan County (MW642407.1)
South Island NZL (MW642406.1)
Okayama JPN (MW642360.1)
Guidong County (MW642347.1)
Queensland AUS (MW642366.1)
New South Wales AUS (MW642364.1)
Shengsi County 2 (MW642397.1)
Yuexi County (MW642389.1)
Kaizhou District (MW642350.1)
Penglai City (MW642362.1)
Changdao County (MW642340.1)
Zhuanghe City (MW642357.1)
Cangxi County (MW642339.1)
Xuyi County (MW642368.1)
Jeju KOR (MW642405.1)
Penglai City 2 (MW642384.1)
Liangdang County (MW642369.1)
New Jersey USA (MW642404.1)
Tengchong City (MW642390.1)
Suichuan County (MW642385.1)
High tech District (MW642372.1)
Hanshan County (MW642348.1)
Hongan County (MW642403.1)
Penglai City 3 (MW642382.1)
Lvshunkou District 2 (MW642402.1)
Macheng City (MW642359.1)
Chaotian District (MW642342.1)
Songjiang District (MW642363.1)
Guangshan County (MW642376.1)
Chaoyang District (MW642375.1)
Penglai City 4 (MW642383.1)
Huoqiu County (MW642338.1)
Changdao County 2 (MW642374.1)
Dinghai District (MW642343.1)
Lvshunkou District 3 (MW642381.1)
Zhuanghe City 2 (MW642399.1)
Jizhou District (MW642398.1)
Pinggu District (MW642400.1)
Daishan County (MW642396.1)
Shijingshan District (MW642392.1)
Danjiangkou City (MW642395.1)
Luoshan County2 (MW642391.1)
Jishan County (MW642336.1)
Lingchuan County (MW642354.1)
Jiangxia District (MW642394.1)
Anji City (MW642393.1)
Mentougou District (MW642358.1)
Jingxing County (MW642337.1)
Dingxiang County (MW642371.1)
Pingshan County (MW642365.1)
Liangdang County 2 (MW642355.1)
Ganquan County MW642345.1)
Fuping County (MW642344.1)
Xin County (MW642367.1)
Oita JPN (MW642361.1)
Yixing City (MW642356.1)
Kuandian County (MW642352.1)
Jinzhou District (MW642379.1)
Hunchun City (MW642378.1)
Kuandian County 2 (MW642370.1)
Huairou District (MW642377.1)
Fengcheng City 2 (MW642373.1)
Xinglong County (MW642386.1)
Miyun District (MW642380.1)
Xiuyan County (MW642388.1)
Laizhou City 3 (MW642353.1)
Anji City 2 (MW642349.1)
Laizhou City (MW642351.1)
Gongyi City (MW642346.1)
Chaohu City (MW642341.1)
Fengcheng City (MW642401.1)
Xinglong County 2 (MW642387.1)
H. hystricis (NC_039765.1)
H. flava (NC_005292.1)
triploid (parthenogenetic)
diploid (bisexual)
0.10
